# The Book World of Medicine and Science

**Published:** 1907-01-19

**Authors:** 


### THE BOOK WORLD OF MEDICINE AND SCIENCE.

TUBERCULOSIS; ITS ORIGIN AND EXTINCTION. By W. PICKETT TURNER, M.D. (London: Adam and Charles Black. 1906. Small 8vo. Pp. 96. Price not stated.)

DR. TURNER reminds us that in England there are always 300,000 people afflicted with consumption, and that the deaths from this disease amount to 60,000 annually; and he argues, perhaps a little illogically, that "Nature could never have intended such an amount of suffering to occur." Nevertheless a disease which claims such a terrible death-roll must ever possess the greatest interest to us all; and medical men are bound to study whatever is presented to them as purporting to throw fresh light on the subject, provided the message comes in reasonable guise. At the onset we may say that Dr. Turner does not believe in any of the serum cures, and although we think he is a little too sweeping in his condemnation of these, more especially as he accepts the doctrine of phagocytosis on which these serum cures are based, he is unquestionably right in protesting against the publicity given by the lay Press to "cures" of which the potency has not yet been demonstrated. Neither do we think the author is right in his estimate of the open-air treatment, although it is quite possible that more was at one time claimed for it than experience has been able to confirm. This is by the way; and we come to Dr. Turner's own thesis. He forms a special group of diseases which he names the "Mycotic" group. These diseases are caused by herbivora eating certain grasses, which have been attacked by some kinds of bacteria. The animals eating these grasses not only suffer themselves but they communicate the disease to others. Tuberculosis is one of these diseases. It is caused by cattle eating grasses on which the tubercle bacillus is present. These cattle are, in Dr. Turner's words, "the intermediate indispensable host"; and man derives the disease from the flesh or milk of such animals. The theory is thus beautifully simple, and the statistics quoted by the author tell us that of 22,918 cattle slaughtered over a year old, 7,619, or 33.21 per cent., were tuberculous. Sheep were very little affected, and goats not at all, while pigs suffered only to the extent of 2.73 per cent. Now it is quite certain that sheep and goats as a rule do not consume the same kind of grass as cattle, and that they spend more time in the open air than cows. In the Island of Jersey, cattle are almost entirely free from tuberculosis, and this tells in favour of Dr. Turner's hypothesis. Another point is that if Dr. Turner is right, English vegetarians ought to suffer less from phthisis than English beef-eaters. Possibly the number of vegetarians may be too small to generalise from; but we venture to suggest the investigation of this point to Dr. Josiah Oldfield. We regret that we have not space to refer to Dr. Turner's suggestions for the prevention of tubercle in animals, as well as many other interesting points, and we give the author's own recapitulation: "Tuberculosis is an animal disease and is primarily derived from cattle. It belongs to the mycotic group of diseases, bovines obtaining it from grasses by natural affinity. Man acquires the disease by ingestion or inoculation, never by inhalation. It is not hereditary, neither is there any predisposition to it in the individual. The bacillus in a state of nature is a saprophyte, but becomes pathogenic in cattle when deprived of actinism."

HUMAN PHYSIOLOGY (First Stage) (Organised Science Series). G. NORMAN MEACHEN, M.D., M.R.C.P. (University Tutorial Press. 2s.)

THIS book is said to have been written to meet the requirements of candidates of the Board of Education, and is intended merely as an introduction to the subject of physiology. In the preface the author says that a modern writer has said that "books on science are often sadly dull," and that an attempt has been made to relieve this book from such a stigma. The style of the author is certainly easy and simple, and, with the aid of the illustrations, anyone desiring to gain an elementary knowledge of physiology should have no excuse for saying that this book does not supply his needs. To combine what we call interest, however, with the necessary number of detailed facts required for examination purposes is not always easy; yet the study of physiology is one that most people will approach with a certain degree of interest, because we are nearly all of us anxious to know something about ourselves. In addition to reading, students are recommended to dissect a rabbit under the supervision of a teacher, and to perform some simple experiments, such as that of the changing of starch into sugar by saliva, and the digestion of proteids by Benger's *liquor pepticus*. Examination questions are given at the end of each chapter, and by means of these the student may test his knowledge.

WELLCOME'S PHOTOGRAPHIC EXPOSURE RECORD AND DIARY. (London: Burroughs Wellcome and Company.)

THIS diary is one more evidence of the business acumen and up-to-dateness of the methods pursued by this firm. It contains much useful information of value to those photographers who have the camera in habitual use in institutions or for their own purposes. The information is full and interesting, and the mechanical calculator and light tables display much ingenuity and render the estimation of the correct exposure for any subject an easy matter. Two diaries are issued, one for the Northern Hemisphere and the other for the tropics and Southern Hemisphere. The diary includes a folding card for hanging up, which conveys much useful information of development by the factorial method.

"WHO'S WHO," 1907. (London: A. and C. Black.)

THIS book continues to grow in size and utility. The information it contains is well up to date, and much of it has an abiding interest for those who have a sense of humour. What can surpass George Bernard Shaw's recreation, "Anything except sport," or his definition of exercise as "Public speaking"? The book is now indispensable as the most useful address directory for office use.

"WHO'S WHO YEAR-BOOK," 1907. (London: A. and C. Black.)

THIS contains the tables which formed the original nucleus of "Who's Who." Its object is to show all the information it contains at a glance, and for busy people of observation it fulfils a distinctly useful purpose.

